# On the Treatment of Puriform Discharge from the Ear

**Published:** 1819-05

**Authors:** J. H. Curtis

**Affiliations:** Surgeon-Aurist to the Prince Regent.


*For the London Medical and Physical Journal.*

### *On the Treatment of Puriform Discharge from the Ear;* by J. H. CURTIS, Esq. Surgeon-Aurist to the Prince Regent.

OF the diseases of the ear, one of the most obstinate and perplexing to practitioners is that attended with a puriform discharge, and so termed from this circumstance. The success of the practice I have adopted in this disagreeable and obstinate complaint has, I believe, been more fortunate than that obtained from the measures generally employed, if we may be allowed to form conclusions from the statements of the best authors on this subject. This cannot be better illustrated than by the recital of a few cases selected from the records of my practice, in the order in which they have occurred.

Miss B. of St. John-street, aged 26, had been from her childhood affected with deafness and a puriform discharge from one ear. After having been under the care of several eminent surgeons in London for upwards of two years without relief, she applied to me. On inspecting the ear, I found the meatus much excoriated by the discharge, which was very profuse and offensive; the tympanum, I observed, was partly destroyed, as air could be forced out of the passage. In other respects the ear appeared perfectly sound. Adopting my usual plan of not stopping the discharge hastily, I ordered a blister to be applied behind the ear, which was kept open for a fortnight; after which the patient used an injection of zinci sulphas; but this not appearing to have the desired effect, I had recourse to the argentum nitratum, as recommended by Mr. Saunders in cases of this nature. The patient began by using ten grains in four ounces of water, and I increased it to the extent of thirty-five grains, which completely healed the parts; and I had the farther satisfaction, at the same time, to find her hearing restored. It may be necessary, perhaps, to mention that it took nine months to complete the cure.

A similar case of a young gentleman yielded also to this treatment; but his case, though not of so long standing, I at first sight considered more difficult of cure, from his having a polypus extending directly across the meatus, which I removed by an instrument I had constructed for the purpose. Immediately after the operation he was able to hear, but the discharge continued for some time, though at last it was happily entirely suppressed.

Another case of the same nature occurred in a lady, who was sent to me by a physician, whose hearing was defective in both ears, in consequence of a puriform discharge. This case yielded in a short time to a varied combination and change of injections, consisting of solutions of zinci sulphas, plumbi superacetas, argentum nitratum, cupri sulphas camph. joined occasionally with camphor and opium.

A great number of cases of a like description have come under my observation, as already stated, at the Royal Dispensary, and have yielded to a similar plan properly persevered in for a length of time.

The above cases, I trust, will sufficiently prove that diseases of this nature are frequently curable, when a proper plan of proceeding is persevered in.

2, *Soho-square; Nov.* 5, 1818.

